# RNA Secondary Structure Modulates FMRP’s Bi-Functional Role in the MicroRNA Pathway

**DOI:** 10.3390/ijms17060985

**Published:** 2016-06-22

**Authors:** Phillip Kenny, Stephanie Ceman

**Affiliations:** 1 Cell and Developmental Biology, University of Illinois-Urbana Champaign, Urbana, IL 61801, USA; pjkenny2@illinois.edu; 2 College of Medicine, University of Illinois-Urbana Champaign, Urbana, IL 61801, USA; 3 Neuroscience Program, University of Illinois-Urbana Champaign, B107 Chemical Life Sciences Laboratory, Urbana, IL 61801, USA

**Keywords:** FMRP, G-Quadruplex, microRNA, MOV10, RNA binding proteins, secondary structure

## Abstract

MicroRNAs act by post-transcriptionally regulating the gene expression of 30%–60% of mammalian genomes. MicroRNAs are key regulators in all cellular processes, though the mechanism by which the cell activates or represses microRNA-mediated translational regulation is poorly understood. In this review, we discuss the RNA binding protein Fragile X Mental Retardation Protein (FMRP) and its role in microRNA-mediated translational regulation. Historically, FMRP is known to function as a translational suppressor. However, emerging data suggests that FMRP has both an agonistic and antagonistic role in regulating microRNA-mediated translational suppression. This bi-functional role is dependent on FMRP’s interaction with the RNA helicase Moloney leukemia virus 10 (MOV10), which modifies the structural landscape of bound mRNA, therefore facilitating or inhibiting its association with the RNA-Induced Silencing Complex.

## 1. Introduction

The clinical consequence of a loss in functional Fragile X Mental Retardation Protein (FMRP) is known as Fragile X Syndrome (FXS), the most common inherited form of intellectual disability [1,2]. Along with impaired cognition, FXS was shown to exist in high comorbidity with Autism Spectrum Disorders (ASD) [3,4]. FMRP is an RNA-binding protein [5], binding approximately 4% of mRNAs in the brain [6]. FXS is, in large part, due to a CGG repeat expansion in the 5′ Untranslated Region (5’ UTR), leading to hypermethylation of the CG dinucleotides including the promoter and ultimately causing the transcriptional silencing of the *FMR1* gene [7]. However, there are a small number of individuals with FXS that have normal CGG repeat numbers but have missense mutations in the *FMR1* coding region. Two identified missense mutations are the arginine to glutamine mutation (R138Q) in the Nuclear Localization Sequence (NLS) [8], also referred to as the homology to hnRNP K0 (KH0) domain [9,10] as well as an isoleucine to asparagine (I304N) mutation in the second KH RNA binding domain (KH2) [11]. Such individuals highlight the importance of the FMRP domains in which these mutations are located and will be discussed in more detail later.

The evidence that FMRP is a translational regulator has long been known, but the mechanisms for this regulation are poorly understood. Two factors that may influence FMRP’s role in the microRNA (miRNA) pathway are the structural landscape of the 3’ Untranslated Region (3′ UTR) and FMRP’s interaction with other RNA-binding proteins (RBPs). Here we discuss the RNA G-Quadruplex (GQ) and FMRP’s interaction with the RNA helicase Moloney leukemia virus 10 (MOV10). The bi-functional role of FMRP to suppress or de-repress its target transcripts through the miRNA pathway may be, in part, through modulating RNA secondary structure through its interaction with MOV10.

## 2. Fragile X Mental Retardation Protein (FMRP), Structure and Function

**Figure 1:**
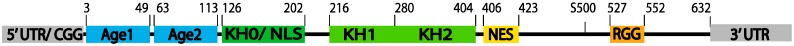
Domain Map of the Fragile X Mental Retardation Protein (FMRP). The domain map of FMRP, displaying its three RNA binding domains: KH1 and KH2 (light green), and arginine-glycine-glycine (RGG) box (orange), two Agenet domains (blue), a newly discovered KH0 domain (dark green) containing the Nuclear Localization Sequence (NLS), the Nuclear Export sequence (NES) (yellow), the 3′ UTR (gray) and 5′ UTR containing the CGG trinucleotide repeat (gray). Also depicted is Serine 500 (S500) (mus499), the critical site of phosphorylation.

Though expressed in many tissue types, FMRP is highly expressed in the testes and the brain, with elevated expression in the cortex and hippocampus [12,13]. FMRP is alternatively spliced and has many isoforms [5,14,15]. The main isoform of FMRP in mammals contains very conserved functional domains, including three RNA-binding motifs [16] (Figure 1). Two of the RNA-binding domains are KH1 and KH2. Though the RNA substrate that KH1 binds is unknown, systematic evolution of ligands by exponential enrichment (SELEX) established that the KH2 binding domain binds a double stem loop structure known as the kissing complex *in vitro*. Although not specifically identified in any cellular RNAs, exogenously introduced kissing complex was able to compete FMRP off of polyribosomes [17], suggesting a functional role. Identification of FMRP binding sites in cells using photoactivatable ribonucleoside enhanced cross-linking immunoprecipitation (PAR-CLIP) suggest there may be two enriched sequences that FMRP binds, ACUK (where “K” = G or U) and WGGA (where ”W“ = A or U) [18]. However, a re-analysis of the PAR-CLIP data suggested that only WGGA was enriched in FMRP target sites [19]. The third RNA-binding domain is the arginine-glycine-glycine (RGG) box, thought to bind G-rich secondary structures, such as G-Quadruplexes (GQs), with nanomolar affinity [20,21,22]. Despite substantial literature describing FMRP binding mRNAs in their 3′ UTR, including its own 3′ UTR [23,24,25], FMRP binding data obtained by high-throughput sequencing of RNAs isolated by crosslinking immunoprecipitation (HITS-CLIP) suggested that FMRP binds predominantly in the coding regions of the mRNA. Surprisingly, the reads obtained did not show enrichment for G-rich secondary structure [26]. More recently, X-ray crystallography analysis revealed that the RGG peptide was shown to bind to specific sites of the GQ through shape complementation, electron rich π-interaction with the potassium cation, and through hydrogen bonding. Importantly, evidenced by this work, the RGG peptide appeared to stabilize the G-tetrads and facilitated GQ formation [27]. Thus, although GQs may not be enriched in the target RNAs bound by FMRP as identified by CLIP, there seems to be a significant role for either a transient interaction of FMRP with GQs or an association of FMRP with GQs in a small subset of RNAs.

Another KH domain was recently discovered and appropriately named the KH0 domain, as it resides N-terminal to the first KH domain (KH1) [9,10]. Although there is no known RNA substrate for KH0, a single point mutation (R138Q) within this domain led to a patient exhibiting the FXS phenotype [28]. However, this mutation did not affect the ability of FMRP to bind a subset of neuronal mRNA targets and is thought to effect FMRP’s protein-protein interactions, specifically those involved in the large-conductance calcium-activated potassium (BK) channel binding [9,29].

FMRP contains two N-terminal in-tandem agenet domains, Agenet1 and Agenet2, thought to associate with trimethylated lysine [30]. Other functions of FMRP in the nucleus were established when it was found that FMRP interacted with methylated H_3_K_79_ through its agenet domains and modulated the DNA repair response [31]. A loss of FMRP resulted in incomplete chromosome pairing and other meiotic defects in mice [31]. Evidence suggests that the agenet domains, comprised of the Tudor, Malignant Brain Tumor Domain (MBT), and chromo domains [32] is a domain involved in FMRP’s protein-protein and protein-RNA interactions [33,34].

Though mostly cytoplasmic, FMRP contains both nuclear localization and export sequences and has been shown to shuttle into the nucleus to bind target mRNAs, eventually transporting back into the cytoplasm with its bound mRNA through the nuclear RNA export factor 1 (Tap/NXF1) [35]. Once translationally suppressed, these Ribonuclear proteins (RNPs) are sequestered into the RNA granules through an unknown mechanism. FMRP also contains a low complexity domain (LCD), a domain containing little amino acid diversity, being enriched in glycine, tyrosine, and serine residues [36]. Low complexity domains are intrinsically disordered and unstructured in solution, and therefore may be involved in flexible binding interactions [37]. Recent work suggests that the interaction of this domain with other RNPs containing LCDs could lead to a dynamic hydrogel-like aggregation, possibly the basis for how RNA granules may form [36].

## 3. FMRP as a Translation Suppressor

FMRP was identified as associating with the 60S large ribosomal subunit in 1996, although the functional consequences of that association were unknown [38]. The first evidence that FMRP functioned as a translational suppressor was shown when incubation of purified recombinant FMRP with RNAs suppressed their translation in *Xenopus* oocytes and in rabbit reticulolysate [39]. Later work showed that FMRP is phosphorylated and that this modification coincided with stalled ribosomes [40]. In a large scale HITS-CLIP analysis of FMRP-mRNA targets, FMRP was shown to stall ribosomal translocation on identified brain RNA, resulting in translationally suppressing these transcripts [26]. Recent work using cryo-electron microscopy revealed that FMRP binds directly to the L5 protein of the 80S subunit of the ribosome, blocking access of transfer RNA (tRNA) and elongation factors and subsequently inhibiting translation [41]. However, the mechanisms by which this interaction could be reversed are unclear. The aforementioned experiment was done with the *Drosophila* ortholog of FMRP, as was much of the initial work implicating FMRP in the RNA-induced silencing complex (RISC) pathway, discussed in more detail below. *Drosophila* has one fragile X family member, whereas mammals have three (FMRP, FXR1P and FXR2P), which are 60% identical [42]. Thus, experiments with the *Drosophila* ortholog likely give insight into the function of all of the fragile X family members. Like FMRP, loss of expression of the *Drosophila* ortholog result in viability but impaired behavior [43].

An alternative mechanism in which FMRP suppresses the translation of its bound mRNAs is by modulating the interactions required for translation initiation. Cytoplasmic FMRP Interaction Protein (CYFIP1) directly interacts with FMRP, with its bound mRNA, along with the eukaryotic initiation factor 4E (eIF4E), sequestering it into an RNP and effectively suppressing cap dependent translation initiation [44]. Translational suppression was found to be reversible upon the stimulation of brain-derived neurotrophic factor (BDNF) receptors (TrkB) or group I metabotropic glutamate receptors (mGluRs) [44], and is dependent upon the phosphorylation of eIF4B by Mitogen Activated Protein (MAP) Kinase-interacting kinase (MNK1), which disrupts CYFIP1-eIF4E association and facilitates translation initiation [45,46]. Thus, FMRP can suppress translation at initiation, elongation and in the 3′ UTR. In neurons, FMRP plays a role in establishing synaptic plasticity by translationally suppressing its bound mRNA at dendritic spines [47,48]. FMRP may also suppress translation through association with the RNA-induced silencing complex (RISC), which will be addressed in more detail below [49,50].

## 4. The Role of FMRP in MicroRNA (miRNA)-Mediated Translational Regulation

The first evidence of FMRP’s role in the RNA interference (RNAi) pathway came from studies in which the *Drosophila* ortholog of FMRP was found to be associated with components of RISC, and regulated mRNA expression through an unknown mechanism [51,52]. In humans, FXR1 and FXR2 enhance [53] or suppress [54] expression of bound mRNA either independently or through their direct interaction with FMRP. Recent work has provided evidence that FMRP acts as a molecular switch for translation, interacting with CYFIP1 to suppress translation initiation or FXR2 to enhance translation of the post synaptic density protein 95 (PSD-95) mRNA [53]. Subsequently, FMRP’s involvement in RNAi in mammals was characterized through its direct interaction with Argonaute in RISC-associated translational regulation as well as in miRNA biogenesis, established through its association with Dicer [55,56]. FMRP-associated mRNAs targeted for translational suppression in yeast were found to localize in cytoplasmic foci termed processing bodies (P-bodies) [57,58]. Along with FMRP, many components of the translational silencing machinery, including Argonaute and GW182 are found within these P-bodies [59]. FMRP was also shown to directly interact with Argonaute2 (AGO2) [60] and this FMRP-RISC Ribonuclear protein complex, containing miR-125b, was found to translationally suppress NR2A, a NMDA receptor subunit in mouse brain [61]. Soon after, metabotropic glutamate receptor (mGluR) signaling was found to facilitate FMRP’s association with AGO2. Phosphorylation of FMRP provides a mechanism for modulating its association with AGO2, demonstrated by the mGluR-stimulated facilitation of miRNA-mediated translational suppression of PSD-95 mRNA, a major neuronal protein [49]. PSD-95, modulated through FMRP’s phosphorylation status, was translationally suppressed by miR-125a [49]. Phosphorylated FMRP was found to recruit the miR-125a-AGO2 inhibitory complex to the 3′ UTR of PSD-95, suppressing translation. DHPG (a potent agonist of group I mGluRs) activation of mGluR was found to facilitate de-phosphorylation of FMRP, leading to dissociation of the inhibitory FMRP-RISC-miR-125a RNP, thereby activating translation [49]. However, the molecular mechanism by which FMRP’s recruitment of RISC to microRNA recognition elements (MRE) embedded within un-resolved RNA secondary structure and its consequent regulation by the miRNA pathway is poorly understood. Recent work involving FMRP’s interaction with other RNA binding proteins and the secondary structure of its bound mRNA may shed light on this question. FMRP was shown to directly interact with the RNA helicase MOV10, another protein implicated in the miRNA pathway [62,63]. MOV10’s binding to target mRNA is facilitated by FMRP, possibly unwinding secondary structure and enhancing accessibility of RISC to once protected MRE sites on target mRNA [50].

## 5. RNA Secondary Structure and Its Effect on miRNA Translational Suppression

The 3′ UTRs of RNA contain excessive secondary structure and are heavily bound by RBPs [64], which can influence how mRNA is regulated in the miRNA pathway. For example, Pumilio1 (PUM1) enhances miR-221 and miR-222 accessibility on the 3′ UTR of p27 mRNA. The MREs for these miRNAs are within a stem loop structure, inaccessible to RISC. Upon growth factor stimulation, phosphorylated levels of PUM1 increase, remodeling and disrupting the stem loop structure to reveal the MRE [65]. As a consequence of opening up secondary structure, the MRE is accessible to RISC, therefore suppressing translation of p27 [65,66].

There is evidence that proximal binding of RBPs to MRE sites may act to block MREs, restricting the accessibility of RISC on target mRNAs and therefore up-regulating the expression of these genes. For example, dead end 1 (Dnd1) competitively binds to 3′ UTR MRE sites in human cell lines and zebra fish germ cells, prohibiting miRNAs from binding, counteracting translational suppression of target mRNA [67]. The Coding Region Determinant- binding Protein (CRD-BP) was shown to bind the 3′ UTR of Microphthalmia-associated Transcription Factor (MITF) mRNA and prevent the binding of miR-340, thereby stabilizing the transcript and causing elevated expression of the protein, a strong regulator of melanogenesis [68]. Very recent work has shown that an isoform of Rbfox1, localized to the cytosol, antagonizes miRNA-mediated translational suppression by blocking MREs within the 3′ UTRs of many genes found to be misregulated in Autism Spectrum Disorders (ASD) [69].

RNA-binding proteins have also been shown to have both an agonizing or antagonizing function in the miRNA-mediated translational regulation of their bound mRNAs. The polypyrimidine tract binding protein (PTB) functions to antagonize miRNA-mediated translational regulation by competing with miRNA targets in the 3′ UTRs of a subset of mRNAs. In this case, partial overlap of the MRE sequence to that of the PTB binding site causes direct competition for this region, decreasing RISC’s ability to bind. Contrary to competing for binding sites with the miRNA, higher concentrations of PTB also facilitate miRNA targeting by modifying secondary structure to reveal or expose MREs [70]. In this subset of mRNA, enhanced PTB binding increases the propensity of a stem loop containing MRE sites for the microRNAs Let-7b, miR-181b, and miR-196a toward single stranded RNA, thereby exposing and enhancing access of RISC to MREs [70].

Another RBP exhibiting both agonistic and antagonistic functions in miRNA regulation based on RBP binding and secondary structure is human antigen R (HuR). HuR facilitates miRNA binding in the 3′ UTR of the c-Myc mRNA, by either recruiting Let-7a or by changing the local structure proximal to the MRE [71], thereby enhancing RISC binding and activating miRNA-mediated translational suppression. However, evidence from work in the same lab showed that HuR antagonized miRNA-mediated suppression by miR-548c-3p by oligomerizing along the 3′ UTR and blocking RISC association with MREs on the DNA Topoisomerase 2 alpha (TOP2A) mRNA [72] or causing dissociation of the miRISC as seen in the cationic amino acid transporter 1 (CAT-1) mRNA [73].

RNA secondary structure itself has a large effect on the efficacy of miRNA function [74]. The modification of secondary structure may be a mechanism in which another level of regulation to either suppress or facilitate miRNA-mediated translational suppression on mRNA can occur. A common RNA secondary structure found in transcripts is the GQ, mentioned above, as being recognized by the RGG box of FMRP. GQs are nucleic acid structures formed by stacked G-quartets (also called G-tetrads), which are square coplanar arrays of four guanines. The four guanines are arranged in a planar conformation that is stabilized by Hoogsteen-type hydrogen bonds [75,76]. Two or more G-quartets stack to form the GQ [77], a structure that is described as one the most thermodynamically stable nucleic acid structures in nature [78,79].

In the 5′ UTR of RNA, GQs have been shown to suppress translation by blocking translation initiation [80,81]. Within the 3′ UTR, MRE sites can be embedded within GQs [20,75]. Argonaute with its bound miRNA (RISC) does not exhibit helicase activity and cannot unwind RNA secondary structure [82]. However, studies have shown that RISC is kinetically able to find its desired MRE site approximately a magnitude faster than free miRNA [82,83]. One factor that increases the ability of RISC to find MRE sites on target mRNA is due to the change in the conformation of the miRNA-AGO2 RNP to a structure that is more thermodynamically prone to binding. A second factor that plays a large role in faciliting RISC binding is the necessity of unfolding secondary structures to allow access of the miRNA-AGO2 RNP to MRE sites [74]. How GQs can regulate the ability for RISC to bind to a substrate was demonstrated in the regulation of the mRNA of PSD-95. The MRE site targeted by miR-125a is embedded within two or more alternating GQ structures. At equilibrium, the more common and preferred structure is the more stable GQ, which exposes the seed sequence AGGGA of miR-125a. The exposed MRE allows the access of RISC, resulting in translational suppression of PSD-95 mRNA. However, the alternate GQ structure embeds the MRE within the GQ, inhibiting the access of RISC and resulting in the de-repression of the mRNA [84]. The more stable GQ structure was found to be independent of potassium concentration while the second stable GQ was much more dynamic and was dependent on potassium concentration [84]. Thus, translational suppression by miR-125a was found to be dependent on which GQ structure was predominant and suggested that it could be modulated via potassium concentration or other factors such as unwinding and refolding by RBPs and/ or RNA helicases [50,84].

## 6. Resolving RNA Secondary Structure: The Role of Moloney Leukemia Virus 10 (MOV10) and RNA Helicases in miRNA Function

RNA helicases function to remodel and unwind RNA and RNA-protein complexes (RNP), and play a role in many different processes. Because secondary structure plays such a significant role in miRNA functional efficiency, it stands to reason that resolving secondary structure is paramount for miRNA-mediated translational regulation. Examples of how RNA helicases function to unwind GQs and enhance or suppress a cellular process is observed by work involving the DEAH-box RNA helicases RNA associated with AU-rich element (RHAU) and DEAH Box Helicase 9 (DHX9). RHAU’s function to unwind GQs [85] has been shown to be necessary in spermatogonia differentiation in mice [86]. DHX9 has been shown to resolve G-rich R-loops, implicated in inducing genetic instability, *in vitro* [87].

**Figure 2:**
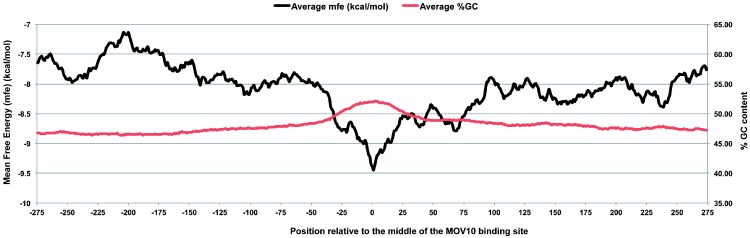
Global analysis of Moloney leukemia virus 10 (MOV10) CLIP sites in the 3′ UTR show an elevated G-rich secondary structure. Mean free energy plot of MOV10 3′ UTR iCLIP sites. Mean free energy of folding ΔG_folding_ was calculated across 55 nt windows ± 275 nt from center of iCLIP site (black). Guanine-cytosine (GC) content (%) is plotted in red [49].

MOV10 is an RNA helicase that binds single stranded RNA proximal to the stop codon and translocates along the 3′ UTR in a 5′ to 3′ direction [88]. Like many RNA helicases that do not appear to have specific binding sequence motifs leading to multiple cellular functions [89], MOV10 has been implicated in contributing to nonsense-mediated decay [88], retrotransposition suppression [90,91] and miRNA-mediated translational regulation. There is evidence that MOV10 may recognize and bind directly to GQs [50]. Global analysis of CLIP-seq data for MOV10 in the 3′ UTR indicated that the region in close proximity to the MOV10 binding sites was predicted to contain a high degree of G-rich secondary structure (Figure 2). However, evidence supporting that MOV10 binds to single stranded RNA proximal to the stop codon and translocates along the 3′ UTR to unwind secondary structure [88] could indicate that the probability of MOV10 at G-rich sites with secondary structure increases as MOV10 requires time to resolve them. Work to establish how MOV10 targets specific mRNA as well as how MOV10 binds and resolves secondary structure is on-going.

MOV10 was first identified as being associated with RISC when it was found to be required for miRNA reporter cleavage and co-localized with AGO2 in P-bodies [63]. A study of MOV10 and AGO2 bound mRNA found a high degree in shared mRNA targets and MOV10’s interaction with AGO2 was RNA-dependent [62]. Recent CLIP-seq studies also indicate a high mRNA target correlation with AGO2 targets, and that AGO2’s association with target mRNA is perturbed in the absence or overexpression of MOV10 [50]. The consquence of these interactions on mRNA fate through the miRNA pathway is discussed below.

## 7. FMRP Modulates an Agonistic or Antagonistic miRNA Function through Its Interaction with MOV10

**Figure 3:**
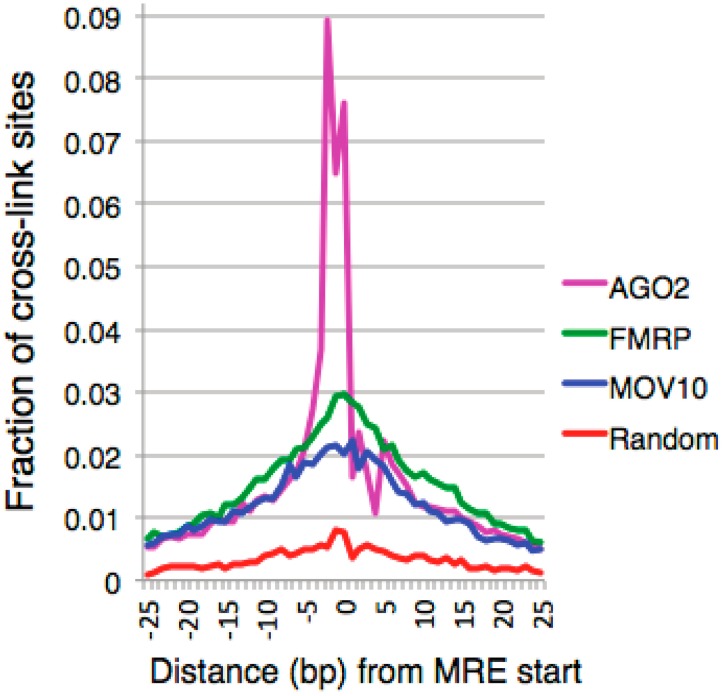
Spatial analysis of CLIP sites of miRNA-associated proteins in the 3′ UTR of target mRNA. Overlay of MOV10 (blue), Argonaute 2 (AGO2) (pink), and FMRP (green) crosslink sites plotted against distance in base pairs to predicted MRE start site. Random sites in random genes were selected as a control (red). The close proximity of CLIP binding sites suggest an interactive role of the proteins on the 3′ UTR of co-bound mRNA [49].

**Figure 4:**
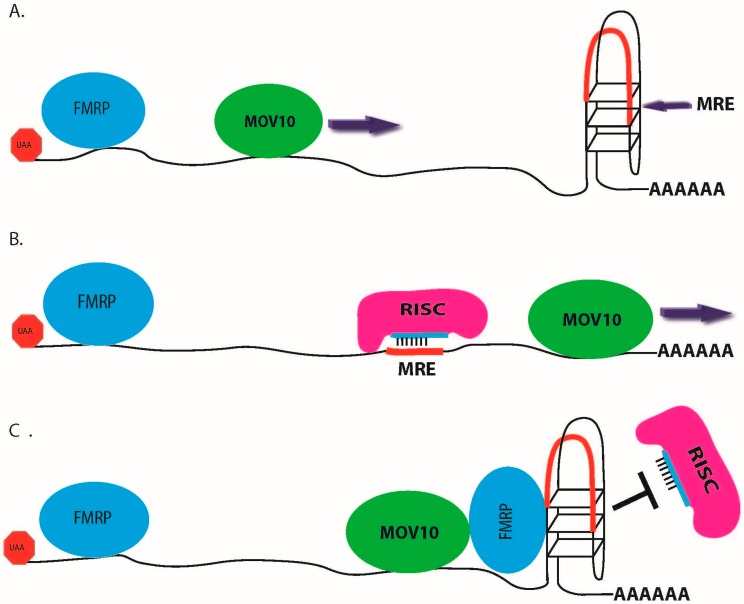
FMRP modulates an agonistic or antagonistic miRNA function through its interaction with MOV10. (**A**) FMRP facilitates MOV10 binding upstream of MREs on the 3′ UTR of co-bound mRNA; (**B**) MOV10 resolves secondary structure to facilitate RISC binding to the MRE site (red) that was once embedded within the GQ; (**C**) FMRP bound proximal to a GQ-containing an MRE acts to block the action of MOV10 and de-represses gene expression.

Analysis of CLIP-seq data for MOV10 [50] AGO2 [70] and FMRP [26] showed that the distance between cross-linked sites for MOV10, FMRP, and AGO2 were not only proximal to one other in the 3′ UTR but also within a short distance to predicted MREs (Figure 3). This suggests an interactive role of these proteins in miRNA-mediated translational regulation. The analysis was performed independent of GC enrichment and therefore consisted of MREs that may or may not be embedded within GQs. Though the exact temporal order of binding in the 3′ UTR of target mRNAs is unknown at this time, FMRP has been shown to facilitate MOV10 binding in our study of MOV10-associated RNAs. In the absence of FMRP, MOV10 had reduced association to co-bound RNAs compared to an elevated association with these RNAs in the presence of FMRP [50]. This observation suggests that FMRP binds RNAs first and then facilitates binding by MOV10—either through its direct interaction with MOV10, thereby affecting the intracellular localization of the complex, or by altering the RNA to make it accessible to MOV10. In most cases, binding by FMRP and MOV10 in the 3′ UTR of mRNA leads to suppression of the mRNA via the miRNA pathway (Figure 4A,B). In this case, FMRP’s recruitment of MOV10 upstream of active MRE sites eventually leads to the re-modeling of the 3′ UTR, exposing MREs and facilitating their interaction with RISC. It is possible that direct interaction of FMRP with AGO2 may also facilitate RISC’s ability to efficiently target MRE sites, thereby facilitating miRNA binding. This interaction would lead to translational suppression. However, when FMRP is bound proximal to MOV10 sites, especially at the site of an MRE embedded in a GQ, RISC association with the mRNA is blocked (Figure 4C). Here, the RNA is protected from miRNA-mediated translational suppression, leading to the elevated expression of these transcripts. A knockdown of FMRP with small inhibitory RNAs (siRNA) segregates a subset of FMRP and MOV10 co-bound mRNAs that showed reduced protein expression levels, based on their spatial binding pattern within the 3′ UTR, suggesting that the antagonistic and agonistic nature of FMRP is dependent on the mRNA and MOV10 [50]. How FMRP’s binding pattern is established and regulated is currently unknown but could be influenced by an increase in its concentration, such as in the case of PTB described previously [70], localization, or by possibly enhancing secondary structure stability, as described by PSD-95 mRNA stability [84]. The mechanism by which FMRP and MOV10 associate at embedded MRE sites, inhibiting MOV10’s ability to resolve secondary structure and attenuating RISC’s association with mRNAs, is currently unknown. Whether this is a cooperative effect in which MOV10 binding facilitates FMRP’s function to stabilize GQ, a result of steric hindrance of the MOV10-FMRP RNP, or the competitive blocking of MOV10’s helicase activity by FMRP is currently unknown and is the focus of future work.

In conclusion, RNA binding proteins and the structural landscape of the 3′ UTR of mRNA play an important role in miRNA translational regulation. It is becoming more apparent that secondary structure not only adds a level of complexity to the dynamic process of miRNA regulation, but the emerging data also supports that the dualistic functions of RBPs can create a multitude of new and exciting methods by which RNA is post transcriptionally regulated. There is still much more work to be done to gain a clearer understanding of how these components work together to cooperatively and/or competitively drive gene expression via the microRNA pathway.
